# Comparison of Robot-Assisted, Laparoscopic, and Open Radical Prostatectomy Outcomes: A Systematic Review and Network Meta-Analysis from KSER Update Series

**DOI:** 10.3390/medicina61010061

**Published:** 2025-01-02

**Authors:** Do Kyung Kim, Young Joon Moon, Doo Yong Chung, Hae Do Jung, Seung Hyun Jeon, Seok Ho Kang, Sunghyun Paick, Joo Yong Lee

**Affiliations:** 1 Department of Urology, Gangnam Severance Hospital, Urological Science Institute, Yonsei University College of Medicine, Seoul 06273, Republic of Korea; 2 Department of Urology, Ewha Womans University Seoul Hospital, Seoul 07804, Republic of Korea; jjunny74@naver.com; 3 Department of Urology, Inha University School of Medicine, Incheon 22332, Republic of Korea; dychung@inha.ac.kr; 4 Department of Urology, Inje University Ilsan Paik Hospital, Inje University School of Medicine, Ilsan 10380, Republic of Korea; haedojung@paik.ac.kr; 5 Department of Urology, Kyung Hee University School of Medicine, Seoul 02447, Republic of Korea; juro@khu.ac.kr; 6 Department of Urology, Korea University College of Medicine, Seoul 02841, Republic of Korea; mdksh@korea.ac.kr; 7 Department of Urology, Konkuk University School of Medicine, Seoul 05030, Republic of Korea; 8 Department of Urology, Severance Hospital, Urological Science Institute, Yonsei University College of Medicine, Seoul 06273, Republic of Korea; 9 Division of Medical Device, Clinical Trials Center, Severance Hospital, Yonsei University Health System, Seoul 03722, Republic of Korea

**Keywords:** prostatic neoplasms, prostatectomy, robotics, laparoscopy, network meta-analysis

## Abstract

*Background and Objectives*: We conducted a systematic review and network meta-analysis to evaluate and compare the perioperative, functional, and oncological outcomes of robot-assisted radical prostatectomy (RARP) and laparoscopic radical prostatectomy (LRP) with open radical prostatectomy (ORP) in patients with prostate cancer. *Materials and Methods:* A comprehensive literature search was performed in Pubmed, EMBASE, and the Cochrane library for papers published before May 2021. Only studies of patients with prostate cancer that assessed perioperative, functional, and oncological outcomes and reported outcome values were included. We used a Bayesian hierarchical random-effects model to synthesize data from multiple studies, enabling both direct and indirect comparisons of the three surgical approaches (RARP, LRP, ORP) to provide robust estimates of their relative efficacy. This systematic review was registered in PROSPERO (CRD42021282555). *Results*: A total of 80 studies were finally included in the present study. Biochemical recurrence (BCR) rates were lower for RARP than for ORP (RR 0.713, 95% CrI 0.587–0.869) and LRP (RR 0.672, 95% CrI 0.505–0.895). Compared with ORP, RARP had a significantly lower positive surgical margin (RR 0.893, 95% CrI 0.807–0.985). When compared to ORP, RARP and LRP showed no significant difference in continence (RR 1.057, 95% CrI 0.997–1.124; RR 0.921, 95% CrI 0.845–1.007). When compared to ORP, RARP was significantly more effective on potency (RR 1.201, 95% CrI 1.047–1.402). The potency rate was significantly higher for RARP than for ORP (RR 1.201, 95% CrI 1.047–1.402) and LRP (RR 1.438, 95% CrI 1.191–1.762). There was no difference in the estimated blood loss or the total and major complication rates between RARP, ORP, and LRP. The operation time was longest for LRP. There was no difference in the operation time between RARP and ORP. *Conclusions*: RARP may be better or comparable to ORP and LRP in terms of oncologic outcomes (PSM and BCR), functional outcomes (potency and incontinence), and perioperative outcomes (EBL, operation time, and total and major complications).

## 1. Introduction

Prostate cancer ranks as the fourth most frequently diagnosed cancer globally, accounting for 7.3% of all new cancer cases in 2022, with an estimated 1.47 million new cases and approximately 396,800 deaths worldwide [1]. The incidence of PCa has increased in recent years in most countries [2]. Currently, men with clinically localized PCa have a variety of treatment options, such as active surveillance, androgen deprivation therapy (ADT), external beam radiation therapy (EBRT), and radical prostatectomy (RP), and RP among several treatment options is the first-line treatment recommended for patients with localized prostate cancer with a life expectancy greater than 10 years [3,4].

Open radical prostatectomy (ORP) is the standard procedure for the treatment of clinically localized PCa; however, this procedure is associated with significant bleeding, postoperative pain, and long hospital stays [5]. Laparoscopic radical prostatectomy (LRP), first reported in the early 1990s, is a minimally invasive procedure that not only has an overall success rate comparable to that of ORP, but also reduces the expected amount of bleeding and shortens the hospital stay after surgery [6]. However, LRP has some limitations, such as uncomfortable postures for the surgeon, two-dimensional imaging, and a steep learning curve due to the high degree of difficulty [7]. Alternatively, robotic-assisted radical prostatectomy (RARP) was introduced in the 2000s, which could overcome the limitations of laparoscopic procedure, thereby lowering the learning curve [7]. RARP has several advantages over standard laparoscopy, including articulated instruments, tremor filtration, and three-dimensional visualization [5]. Therefore, RARP has been widely adopted worldwide as a standard procedure for clinically localized PCa [8].

There have been many studies comparing the perioperative, functional, and oncological outcomes of three surgical procedures (ORP, LRP, and RARP). There are several systematic reviews and meta-analyses comparing RARP, LRP, and ORP by synthesizing the results of these studies [5,9,10]. Cao et al. reported that RARP and LRP are associated with lower blood loss, a lower transfusion rate, and shorter hospitalization durations compared to OPR [5]. However, they conducted the analysis by grouping RARP and LRP into one group. Seo et al. reported that RARP may be beneficial compared to ORP with regard to postoperative complications, perioperative outcomes, and functional outcomes [9]. Carbonara et al. showed that RARP may offer favorable outcomes in potency and continence rates, and less likelihood of biochemical recurrence (BCR) compared with LRP [10]. However, these several systematic reviews and meta-analyses are inconsistent with not only the compared groups, but also the evaluated outcomes. Therefore, we performed a systematic review and network meta-analysis to directly and indirectly compare and evaluate the perioperative, functional, and oncological outcomes of the three surgical procedures (ORP, LRP, and RARP).

## 2. Materials and Methods

This systematic review was registered in PROSPERO (https://www.crd.york.ac.uk/prospero/ (accessed on 31 December 2024), CRD42021282555).

### 2.1. Literature Search

A comprehensive literature search was performed for English-language publications of relevant studies in the PubMed/Medline, Embase, and Cochrane Library databases up to May 2021. The following search terms were included: “prostate cancer”, “prostatectomy”, “laparoscopic”, “robotic”, “open”, and relevant variants. All conference and meeting abstracts, regardless of relevance, were excluded. There were no restrictions on the type of study design. The initial search found 29,959 eligible articles, of which 22,483 studies remained after removal of duplicates. Two authors (DKK and JYL) reviewed the titles and abstracts of 22,483 studies based on inclusion and exclusion criteria, respectively. In case of discrepancy between the two authors, it was decided through discussion whether or not to include the paper in the present study.

### 2.2. Trial Inclusion Criteria and Exclusion Criteria

The eligibility of studies to undertake full-text review was assessed using the PICOS (participant, intervention, comparator, outcome, and study design) approach in accordance with the Preferred Reporting Items for Systematic Reviews and Meta-Analysis (PRISMA) guidelines [11]. The study population included all men with prostate cancer who underwent surgery. The intervention was the three surgical procedures (ORP, LRP, and RARP). The outcomes included oncologic outcomes (BCR and positive margin rate), functional outcomes (continence and potency rate), and perioperative outcomes (estimated blood loss [EBL], operation time, total complications, and major complications).

The following inclusion criteria were applied to the identified studies: human research, men with prostate cancer, patients underwent radical prostatectomy, and studies reported outcome values. Additionally, the following exclusion criteria were applied: studies that compared the cost effect between the types of radical prostatectomy; studies where data could not be extracted; and studies comparing simple prostatectomy.

### 2.3. Data Extraction

Two authors (DKK and JYL) extracted data independently according to a predesigned form. All conflicts in the extracted data were resolved through agreement with the third author (HDJ). The following data were extracted: first author, publication year, study design, country, treatment arms, number of patients, age, surgical technique of the RP, and outcomes (estimated blood loss [EBL], operation time, total complications, and major complications). The functional outcomes included continence and potency. Oncologic outcomes included BCR and positive margin. Perioperative outcomes included estimated blood loss, operation time, major complications, and total complications. Total complications included all reported adverse events during or after surgery, irrespective of severity. Major complications referred to events classified as Clavien–Dindo grade III or higher, requiring surgical, endoscopic, or radiological intervention.

### 2.4. Study Quality Assessments and Quality of Evidence

The designs included in the present study were RCT, prospective, and retrospective designs. Study quality evaluation was conducted for RCT and non-RCT designs.

We assessed the study quality of RCTs based on Cochrane’s assessment of risk of bias that rates aspects of RCT designs [12]. Cochrane’s assessment of risk of bias takes into account factors for random sequence generation, concealment of allocation, blinding of participants and researchers, blinding of outcome assessment, incomplete outcome data, selective reporting, and others.

Quality assessment of non-RCTs was performed using the Newcastle–Ottawa Scale [13]. The Newcastle–Ottawa scale consists of three main rating categories: selection, comparability, and exposure. Stars are awarded to determine quality, with a maximum of 9 stars indicating the highest quality and 6 or more studies indicating high quality.

### 2.5. Statistical Analysis

To indirectly compare the effects of each surgical method of RP, a Bayesian hierarchical random-effects model for outcomes was used for the network meta-analysis. The network meta-analyses were performed using R version 3.4.3 (R development Core Team, Vienna, http://www.R-project.org, accessed on 2 January 2025) with the GEMTC package. All *p*-values were two-sided and a *p*-value < 0.05 was considered statistically significant in all analyses.

We modeled the dichotomous (BCR, continence, complication, positive margin, potency) and continuous outcomes (EBL, op time) for every surgical method in all trials, and comparative effectiveness is reported as the median of posterior distribution of the risk ratios (RRs) and mean differences (MDs) with 95% credible intervals (CrIs) of the studies. Note that a CrI is similar to a conventional confidence interval (CI). Combined estimates were analyzed via the Markov chain Monte Carlo method in which each chain has 20,000 simulations after the first 5000 simulations are discarded as burn-in. To compute the inconsistency of the model, the node-splitting method was applied and 95% CIs of inconsistency factors including zero or a large probability value (*p*-value > 0.05) for the comparison between direct and indirect effects means that there is no significant inconsistency [14]. For example, if studies comparing B–A, C–A, and C–B existed, consistency meant that the effect of B on A plus the effect of C on B equals the effect of C on A [15]. The relative effects were also reported visually using relative effect tables and plots.

## 3. Results

### 3.1. Systematic Review Process

**Figure 1:**
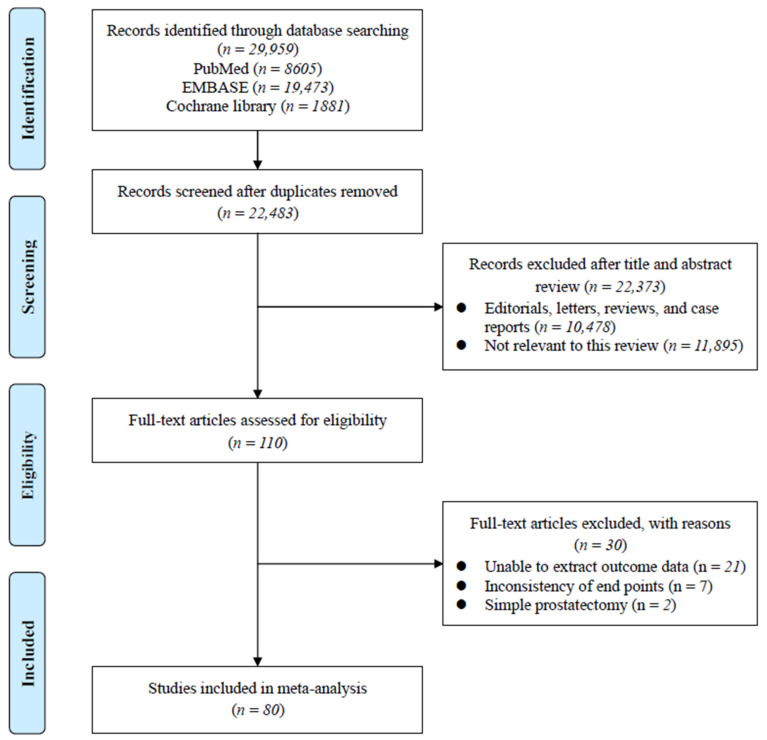
Preferred reporting items for systematic reviews and meta-analysis flowchart. RCT: randomized controlled trial.

The systematic review process of the present study is summarized in the PRISMA flowchart in Figure 1. The initial database search found 29,959 studies, of which 22,483 remained after removal of duplicates. After reviewing the titles and abstracts of the remaining studies, 110 articles remained for full-text review to evaluate compliance with the inclusion and exclusion criteria. Finally, this study included 80 studies with a total of 62,158 patients. Supplement Table S1 shows the characteristics of the included studies.

### 3.2. Bayesian Framework Network Meta-Analysis

#### 3.2.1. Oncologic Outcomes

##### Biochemical Recurrence

**Figure 2:**
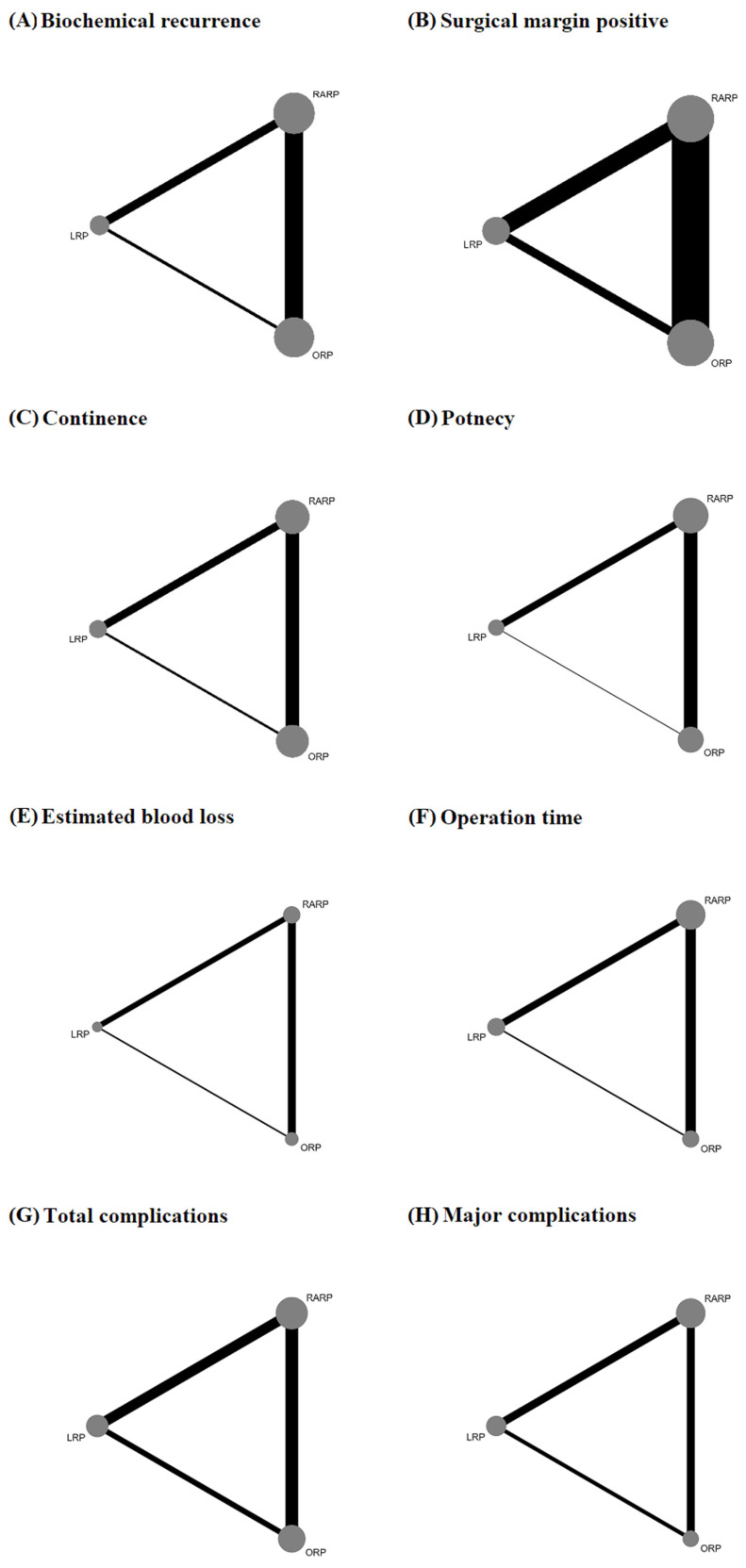
Network plot of the analysis. (**A**) Biochemical recurrence, (**B**) surgical margin positive, (**C**) continence, (**D**) potency, (**E**) estimated blood loss, (**F**) operation time, (**G**) total complications, and (**H**) major complications. The width of the lines is proportional to the number of trials comparing every pair of treatments, and the size of every node is proportional to the number of randomized participants.

This analysis was conducted on 32 studies with a total of 26,368 patients. There were three nodes (RRP, LRP, and RARP) and three comparisons in the network plot (Figure 2A).

**Figure 3:**
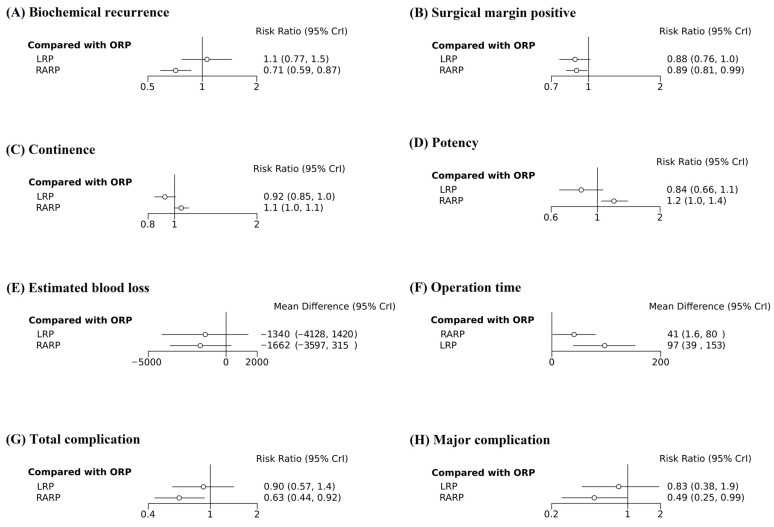
Forest plot of the outcomes. (**A**) Biochemical recurrence, (**B**) surgical margin positive, (**C**) continence, (**D**) potency, (**E**) estimated blood loss, (**F**) operation time, (**G**) total complications, and (**H**) major complications.

The forest plot of the network meta-analysis is shown in Figure 3A. Compared with ORP and LRP, RARP had a significantly lower BCR (RR 0.713, 95% CrI 0.587–0.869; RR 0.672, 95% CrI 0.505–0.895). LRP showed no significant difference in BCR compared to ORP (RR 1.060, 95% CrI 0.771–1.452). The relative effect table reconfirmed these findings (Supplement Table S2). There were no significant inconsistencies in the results in the three node-splitting models.

##### Surgical Margin Positive

This analysis was conducted on 68 studies with a total of 50,232 patients. There were three nodes (RRP, LRP, and RARP) and three comparisons in the network plot (Figure 2B).

The forest plot of the network meta-analysis is shown in Figure 3B. Compared with ORP, RARP had a significantly lower positive surgical margin (RR 0.893, 95% CrI 0.807–0.985). LRP showed no significant difference in positive surgical margin compared to ORP (RR 0.878, 95% CrI 0.756–1.019). The relative effect table reconfirmed these findings (Supplement Table S3). There were no significant inconsistencies in the results in the three node-splitting models.

#### 3.2.2. Functional Outcomes

##### Continence

This analysis was conducted on 30 studies with a total of 17,676 patients. There were three nodes (RRP, LRP, and RARP) and three comparisons in the network plot (Figure 2C).

The forest plot of the network meta-analysis is shown in Figure 3C. When compared to ORP, RARP and LRP showed no significant difference in continence (RR 1.057, 95% CrI 0.997–1.124; RR 0.921, 95% CrI 0.845–1.007). The relative table plot reconfirmed these findings (Supplement Table S4). There were no significant inconsistencies in the results in the three node-splitting models.

##### Potency

This analysis was conducted on 27 studies with a total of 15,333 patients. There were three nodes (RRP, LRP, and RARP) and three comparisons in the network plot (Figure 2D).

The forest plot of the network meta-analysis is shown in Figure 3D. When compared to ORP and LRP, RARP was significantly more effective on potency (RR 1.201, 95% CrI 1.047–1.402, RR 1.438, 95% CrI 1.191–1.762). LRP showed no significant difference in potency compared to ORP (RR 0.836, 95% CrI 0.657–1.065). The relative table plot reconfirmed these findings (Supplement Table S5). There were no significant inconsistencies in the results in the three node-splitting models.

#### 3.2.3. Perioperative Outcomes

##### Estimated Blood Loss

This analysis was conducted on 17 studies with a total of 4375 patients. There were three nodes (RRP, LRP, and RARP) and three comparisons in the network plot (Figure 2E).

The forest plot of the network meta-analysis is shown in Figure 3E. Compared with ORP, RARP and LRP showed no significant difference in estimated blood loss (MD—1662.253, 95% CrI—3597.514–315.091; MD—1340.359, 95% CrI—4128.900–1420.257). The relative effect table reconfirmed these findings (Supplement Table S6). There were no significant inconsistencies in the results in the three node-splitting models.

##### Operation Time

This analysis was conducted on 68 studies with a total of 50,232 patients. There were three nodes (RRP, LRP, and RARP) and three comparisons in the network plot (Figure 2F).

The forest plot of the network meta-analysis is shown in Figure 3F. Compared with ORP, RARP and LRP had a significantly longer operation time (MD 97.092, 95% CrI 39.353–152.799; MD 40.817, 95% CrI 1.615–80.094). The relative effect table reconfirmed these findings (Supplement Table S7). There were no significant inconsistencies in the results in the three node-splitting models.

##### Total Complications

This analysis was conducted on 28 studies with a total of 16,071 patients. There were three nodes (RRP, LRP, and RARP) and three comparisons in the network plot (Figure 2G).

The forest plot of the network meta-analysis is shown in Figure 3G. Compared with ORP, RARP had significantly fewer total complications (RR 0.631, 95% CrI 0.440–0.918). LRP showed no significant difference in positive total complications compared to ORP (RR 0.904, 95% CrI 0.572–1.419). The relative effect table reconfirmed these findings (Supplement Table S8). There was no significant inconsistency in the results in only one node-splitting model out of three. The remaining two node-splitting models showed inconsistency.

##### Major Complications

This analysis was conducted on 19 studies with a total of 11,297 patients. There were three nodes (RRP, LRP, and RARP) and three comparisons in the network plot (Figure 2H).

The forest plot of the network meta-analysis is shown in Figure 3H. Compared with ORP, RARP showed significant fewer major complications (RR 0.493, 95% CrI 0.248–0.995). LRP showed no significant difference in major complications compared to ORP (RR 0.826, 95% CrI 0.380–1.935). The relative effect table reconfirmed these findings (Supplement Table S9). There were no significant inconsistencies in the results in the three node-splitting models.

### 3.3. Quality Assessment and Qualitative Risk of Bias

The risk-of-bias graph and assessment for RCTs are summarized in Supplement Figures S1 and S2. The quality assessment of non-RCTs is summarized in Supplement Table S10. The Newcastle–Ottawa scale ranged from a minimum of 5 to a maximum of 7.

## 4. Discussion

The present study reported the following: (1) RARP was comparable to ORP regarding continence, EBL, operation time, and total complications; (2) RARP showed better outcome in BCR, PSM, potency, and major complications compared to ORP; (3) RARP was comparable to LRP regarding PSM, EBL, total complications, and major complications; and (4) RAPR showed better outcomes in BCR, continence, potency, and operation time compared to LRP. This outcome means that RARP may be better or comparable to ORP and LRP for the treatment of PCa patients.

The main outcomes of RP have been classically reported as trifecta rates indicating the possibility of achieving urinary continence, potency, and cancer control concurrently following surgery [16]. In 2011, Patel et al. introduced the novel concept of “pentafecta”, which is defined as the achievement of potency, continence, BCR-free survival rates, no postoperative complications, and negative surgical margins [17]. They suggested that pentafecta rates could more accurately describe postoperative patient satisfaction because patients eventually want to know if the treatment they receive will leave them cancer-free with minimal complications and the shortest possible recovery time while preserving normal urinary and sexual function. Therefore, pentafecta rates may offer a more comprehensive approach to determining outcomes after RP.

The oncologic outcomes of the present study included BCR and PSM. The definition of BCR depends on the first-line treatment (RP versus first-line treatment radiation therapy) the patient has received [18]. After RP, PSA typically drops to a nadir level, and BCR is defined as two consecutive PSA values higher than 0.2 ng/mL and rising [19]. If BCR occurs within 6 months of RP, it means that metastasis is very likely [20]. PSM in RP specimens has been consistently linked to an increased risk of BCR. There is evidence from several studies that PSM is associated with a higher risk of BCR [21,22,23,24,25]. BCR in men who have received RP with PSM is most likely the result of local recurrence [26]. Therefore, BCR and PSM are very important oncological outcomes for localized PCa after RP, and they are also relevant for adjuvant therapy [5]. This study reported that RARP may have a significantly lower rate of BCR and PSM than ORP and LRP. The role of pelvic lymphadenectomy varies across surgical approaches, and its relevance is pronounced in high-risk prostate cancer. Sentinel node biopsy, which is gaining interest in the urological community, offers complementary benefits when integrated with these approaches. Studies have highlighted the potential of indocyanine green guidance during laparoscopic radical prostatectomy to enhance lymph node dissection efficiency [27,28]. These advancements warrant consideration in future comparative analyses.

After RP was initially introduced in the early 1900s [29], the groundbreaking work by Walsh et al. greatly enhanced the knowledge of prostate surgical anatomy and laid the foundation for the later advancement of the anatomical radical prostatectomy technique [30]. Since early accounts of this procedure, RP has aimed at fully removing the prostate, ensuring optimal cancer control while preserving urinary continence and sexual function [31,32,33]. Consequently, sexual potency and urinary continence are the most critical functional results following RP. However, the findings related to potency and continence should be interpreted with caution, as they are based on smaller datasets drawn from four studies that provided detailed definitions and outcomes for these parameters. Additionally, the definitions of potency and urinary continence varied. Surgical techniques such as nerve-sparing methods, bladder neck preservation, and posterior musculofascial reconstruction were linked to outcomes related to urinary continence and potency [34,35]. These influencing factors could not be excluded or analyzed in subgroups in the current meta-analysis. The role of pelvic lymphadenectomy varies across surgical approaches, and its relevance is pronounced in high-risk prostate cancer. Sentinel node biopsy, which is gaining interest in the urological community, offers complementary benefits when integrated with these approaches. Studies such as those by [doi: 10.1111/iju.14513] and [doi: 10.1016/j.urolonc.2022.08.005] highlight the potential of indocyanine green guidance during laparoscopic radical prostatectomy to enhance lymph node dissection efficiency. These advancements warrant consideration in future comparative analyses. Despite these limitations, it is noteworthy that the results of our study show that RARP is not significantly inferior to, or may even be superior to, other surgical methods in terms of functional outcomes.

Perioperative outcomes compared between RARP, LRP, and ORP in this study included estimated blood loss, operative time, total complications, and major complications. In previous meta-analyses, ORP showed the shortest operative time [5,35], and, similar to their findings, our results indicate that LRP required the most time, followed by RARP, with ORP having the shortest operative time. In our study, there was no difference in EBL (estimated blood loss) between the surgical methods. RARP showed significantly lower rates of total complications and major complications compared to ORP, while there was no significant difference compared to LRP.

The strength of this study is that, unlike previous meta-analyses, it is the first network meta-analysis to indirectly compare all three surgical methods (RARP, ORP, and LRP) simultaneously. Furthermore, it is a very large-scale systematic review and network meta-analysis, including a total of 80 studies with 62,158 patients. However, despite these strengths, the study could not overcome the following limitations. First, only 6 out of the 80 included studies were RCTs, which may result in a lower evidence level. Second, the inclusion of 80 studies introduces high heterogeneity due to factors such as differences in surgical techniques, surgeon skill and experience, and baseline differences in patient characteristics, which may influence the reported results.

## 5. Conclusions

This study showed that RARP may be better or comparable to ORP and LRP in terms of oncologic outcomes (PSM and BCR), functional outcomes (potency and incontinence), and perioperative outcomes (EBL, operation time, and total and major complications). Nevertheless, the majority of the included studies were non-randomized, and there was clear evidence of moderate to significant heterogeneity. Therefore, additional well-designed, multicenter RCTs with extended follow-up periods are necessary to provide stronger evidence.

## Data Availability

No new data were created or analyzed in this study. Data sharing is not applicable to this article.

## Supplementary Materials

The following supporting information can be downloaded at: https://www.mdpi.com/article/10.3390/medicina61010061/s1, Figure S1: The risk of bias graph for RCT; Figure S2: The risk of bias assessment for RCT; Table S1: Characteristics of included studies; Table S2: Relative effect table of operation method’s efficacy for biochemical recurrence; Table S3: Relative effect table of operation method’s efficacy for positive surgical margin; Table S4: Relative effect table of operation method’s efficacy for continence; Table S5: Relative effect table of operation method’s efficacy for potency; Table S6: Relative effect table of operation method’s efficacy for estimated blood loss; Table S7: Relative effect table of operation method’s efficacy for operation time; Table S8: Relative effect table of operation method’s efficacy for total complications; Table S9: Relative effect table of operation method’s efficacy for major complications; Table S10: The Newcastle–Ottawa scale for non-RCT studies.
