# Supplementary material for: Comparison of Robot-Assisted, Laparoscopic, and Open Radical Prostatectomy Outcomes: A Systematic Review and Network Meta-Analysis from KSER Update Series
Source: Medicina (Kaunas). 2025 Jan 2;61(1):61. doi: 10.3390/medicina61010061 (PMC11766646; doi:10.3390/medicina61010061)

1. Search terms

Pubmed

((("prostatic neoplasms"[MeSH Terms] OR ("prostate neoplasms"[Title/Abstract] OR "prostate cancer*"[Title/Abstract] OR "prostatic cancer*"[Title/Abstract] OR "prostate neoplasm"[Title/Abstract] OR "prostatic neoplasm*"[Title/Abstract]) OR "prostate tumo*"[Title/Abstract] OR "prostatic tumo*"[Title/Abstract] OR "prostate carcinoma*"[Title/Abstract] OR "prostatic carcinoma*"[Title/Abstract] OR "prostate malignan*"[Title/Abstract] OR "prostatic malignan*"[Title/Abstract] OR ("prostatectomy"[MeSH Terms] OR "prostatectom*"[Title/Abstract] OR "prostate resect*"[Title/Abstract] OR "prostatic resect*"[Title/Abstract] OR "prostate remov*"[Title/Abstract] OR "prostatic remov*"[Title/Abstract])) AND ("laparoscopy"[MeSH Terms] OR "laparoscop*"[Title/Abstract] OR ("robotics"[MeSH Terms] OR "robotic*"[Title/Abstract]) OR ("robotic surgical procedures"[MeSH Terms] OR ("robotic surgical procedure*"[Title/Abstract] OR "robot assisted surger*"[Title/Abstract])) OR "robotic assisted radical prostatectom*"[Title/Abstract] OR "robot assisted prostatectom*"[Title/Abstract] OR "robotic assisted laparoscopic prostatectom*"[Title/Abstract] OR "robotic assisted"[Title/Abstract] OR "open"[Title/Abstract])) NOT ("animals"[MeSH Terms] NOT "humans"[MeSH Terms])) AND (english[Filter])

EMBASE

(((('prostate neoplasms'/exp OR 'prostate neoplasms':ab,ti) OR ('prostate cancer'/exp OR ('prostate cancer*':ab,ti OR 'prostatic cancer*':ab,ti)) OR ('prostate tumor'/exp OR ('prostate neoplasm':ab,ti OR 'prostatic neoplasm*':ab,ti OR 'prostate tumo*':ab,ti OR 'prostatic tumo*':ab,ti)) OR ('prostate carcinoma'/exp OR ('prostate carcinoma*':ab,ti OR 'prostatic carcinoma*':ab,ti)) OR 'prostate malignan*':ab,ti OR 'prostatic malignan*':ab,ti) OR (('prostatectomy'/exp OR (prostatectom*:ab,ti OR 'prostate resect*':ab,ti)) OR 'prostatic resect*':ab,ti OR 'prostate remov*':ab,ti OR 'prostatic remov*':ab,ti)) AND (('laparoscopy'/exp OR laparoscop*:ab,ti) OR ('robotics'/exp OR robotic*:ab,ti) OR ('robot assisted surgery'/exp OR ('robotic surgical procedure*':ab,ti OR 'robot assisted surger*':ab,ti)) OR ('robot-assisted prostatectomy'/exp OR ('robotic assisted radical prostatectom*':ab,ti OR 'robot assisted prostatectom*':ab,ti)) OR ('robotic assisted laparoscopic prostatectomy'/exp OR 'robotic assisted laparoscopic prostatectom*':ab,ti) OR 'robotic assisted':ab,ti OR open:ab,ti)) NOT ('animal'/exp NOT 'human'/exp) AND [english]/lim

Cochrane library

((prostatic neoplasm)[MeSH Terms] OR ("prostate neoplasms": ab,ti OR (prostate next cancer*): ab,ti OR (prostatic next cancer*): ab,ti OR (prostate neoplasm): ab,ti OR (prostatic next neoplasm*): ab,ti) OR (prostate tumo*): ab,ti OR (prostatic next tumo*): ab,ti] OR (prostate next carcinoma*): ab,ti OR (prostatic next carcinoma*): ab,ti OR (prostate next malignan*): ab,ti OR (prostatic next

malignan*): ab,ti) OR (((prostatectomy)[MeSH Terms] OR (prostatectom*): ab,ti) OR ((prostate next resect*): ab,ti OR (prostatic next resect*): ab,ti OR (prostate next remov*):ab,ti OR (prostatic next remov*):ab,ti)) AND (((laparoscopy)[MeSH Terms] OR (laparoscop*):ab,ti) OR ((robotics)[MeSH Terms] OR (robotic*): ab,ti) OR ((robotic surgical procedures)[MeSH Terms] OR ((robotic next surgical next procedure): ab,ti OR (robot next assisted next surger*): ab,ti)) OR ((robotic next assisted next radical next prostatectom*): ab,ti) OR ((robot next assisted next prostatectom*): ab,ti) OR ((robotic next assisted next laparoscopic next prostatectomy): ab,ti) OR ((robotic assisted): ab,ti) OR ((open):ab,ti))) NOT ((animal)[MeSH Terms] NOT (human)[MeSH Terms])

2. List of studies included in the analysis

[1] Ahlering TE, Woo D, Eichel L, Lee DI, Edwards R, Skarecky DW. Robot-assisted versus open radical prostatectomy: a comparison of one surgeon's outcomes. Urology. 2004;63:819-22.

[2] Asimakopoulos AD, Pereira Fraga CT, Annino F, Pasqualetti P, Calado AA, Mugnier C. Randomized comparison between laparoscopic and robot-assisted nerve-sparing radical prostatectomy. J Sex Med. 2011;8:1503-12.

[3] Barocas DA, Salem S, Kordan Y, Herrell SD, Chang SS, Clark PE, et al. Robotic assisted laparoscopic prostatectomy versus radical retropubic prostatectomy for clinically localized prostate cancer: comparison of short-term biochemical recurrence-free survival. J Urol. 2010;183:990-6.

[4] Breyer BN, Davis CB, Cowan JE, Kane CJ, Carroll PR. Incidence of bladder neck contracture after robot-assisted laparoscopic and open radical prostatectomy. BJU Int. 2010;106:1734-8.

[5] Carlsson S, Nilsson AE, Schumacher MC, Jonsson MN, Volz DS, Steineck G, et al. Surgery-related complications in 1253 robot-assisted and 485 open retropubic radical prostatectomies at the Karolinska University Hospital, Sweden. Urology. 2010;75:1092-7.

[6] Chan C, Chiu AW, Chen M, Hsu JM, Yang S, Lin WR. A comparative study of laparoscopic and robotic assisted radical prostatectomy performed by a single surgeon. Urological Science. 2017;28:71-4.

[7] Chan RC, Barocas DA, Chang SS, Herrell SD, Clark PE, Baumgartner R, et al. Effect of a large prostate gland on open and robotically assisted laparoscopic radical prostatectomy. BJU Int. 2008;101:1140-4.

[8] Choo MS, Choi WS, Cho SY, Ku JH, Kim HH, Kwak C. Impact of prostate volume on oncological and functional outcomes after radical prostatectomy: robot-assisted laparoscopic versus open retropubic. Korean J Urol. 2013;54:15-21.

[9] Coronato EE, Harmon JD, Ginsberg PC, Harkaway RC, Singh K, Braitman L, et al. A multi-institutional comparison of radical retropubic prostatectomy, radical perineal prostatectomy, and robot-assisted laparoscopic prostatectomy for treatment of localized prostate cancer. J Robot Surg.

2009;3:175.
[10] Coughlin GD, Yaxley JW, Chambers SK, Occhipinti S, Samaratunga H, Zajdlewicz L, et al. Robot-assisted laparoscopic prostatectomy versus open radical retropubic prostatectomy: 24-month outcomes from a randomised controlled study. Lancet Oncol. 2018;19:1051-60.
[11] D'Alonzo RC, Gan TJ, Moul JW, Albala DM, Polascik TJ, Robertson CN, et al. A retrospective comparison of anesthetic management of robot-assisted laparoscopic radical prostatectomy versus radical retropubic prostatectomy. J Clin Anesth. 2009;21:322-8.
[12] Dahl DM, Barry MJ, McGovern FJ, Chang Y, Walker-Corkery E, McDougal WS. A prospective study of symptom distress and return to baseline function after open versus laparoscopic radical prostatectomy. J Urol. 2009;182:956-65.
[13] Di Pierro GB, Baumeister P, Stucki P, Beatrice J, Danuser H, Mattei A. A prospective trial comparing consecutive series of open retropubic and robot-assisted laparoscopic radical prostatectomy in a centre with a limited caseload. Eur Urol. 2011;59:1-6.
[14] Doumerc N, Yuen C, Savdie R, Rahman MB, Rasiah KK, Pe Benito R, et al. Should experienced open prostatic surgeons convert to robotic surgery? The real learning curve for one surgeon over 3 years. BJU Int. 2010;106:378-84.
[15] Drouin SJ, Vaessen C, Hupertan V, Comperat E, Misraï V, Haertig A, et al. Comparison of mid-term carcinologic control obtained after open, laparoscopic, and robot-assisted radical prostatectomy for localized prostate cancer. World J Urol. 2009;27:599-605.
[16] Farnham SB, Webster TM, Herrell SD, Smith JA, Jr. Intraoperative blood loss and transfusion requirements for robotic-assisted radical prostatectomy versus radical retropubic prostatectomy. Urology. 2006;67:360-3.
[17] Ficarra V, Novara G, Fracalanza S, D'Elia C, Secco S, Iafrate M, et al. A prospective, non-randomized trial comparing robot-assisted laparoscopic and retropubic radical prostatectomy in one European institution. BJU Int. 2009;104:534-9.
[18] Forsmark A, Gehrman J, Angenete E, Bjartell A, Björholt I, Carlsson S, et al. Health Economic Analysis of Open and Robot-assisted Laparoscopic Surgery for Prostate Cancer Within the Prospective Multicentre LAPPRO Trial. Eur Urol. 2018;74:816-24.
[19] Fracalanza S, Ficarra V, Cavalleri S, Galfano A, Novara G, Mangano A, et al. Is robotically assisted laparoscopic radical prostatectomy less invasive than retropubic radical prostatectomy? Results from a prospective, unrandomized, comparative study. BJU Int. 2008;101:1145-9.
[20] Froehner M, Koch R, Leike S, Novotny V, Twelker L, Wirth MP. Urinary tract-related quality of life after radical prostatectomy: open retropubic versus robot-assisted laparoscopic approach. Urol Int. 2013;90:36-40.
[21] Froehner M, Novotny V, Koch R, Leike S, Twelker L, Wirth MP. Perioperative complications after radical prostatectomy: open versus robot-assisted laparoscopic approach. Urol Int. 2013;90:312-5.

[22] Guazzoni G, Cestari A, Naspro R, Riva M, Centemero A, Zanoni M, et al. Intra- and peri-operative outcomes comparing radical retropubic and laparoscopic radical prostatectomy: results from a prospective, randomised, single-surgeon study. Eur Urol. 2006;50:98-104.
[23] Haese A, Knipper S, Isbarn H, Heinzer H, Tilki D, Salomon G, et al. A comparative study of robot-assisted and open radical prostatectomy in 10 790 men treated by highly trained surgeons for both procedures. BJU Int. 2019;123:1031-40.
[24] Haglind E, Carlsson S, Stranne J, Wallerstedt A, Wilderäng U, Thorsteinsdottir T, et al. Urinary Incontinence and Erectile Dysfunction After Robotic Versus Open Radical Prostatectomy: A Prospective, Controlled, Nonrandomised Trial. Eur Urol. 2015;68:216-25.
[25] Hakimi AA, Blitstein J, Feder M, Shapiro E, Ghavamian R. Direct comparison of surgical and functional outcomes of robotic-assisted versus pure laparoscopic radical prostatectomy: single-surgeon experience. Urology. 2009;73:119-23.
[26] Ham WS, Park SY, Kim WT, Koo KC, Lee YS, Choi YD. Open versus robotic radical prostatectomy: a prospective analysis based on a single surgeon's experience. J Robot Surg. 2008;2:235-41.
[27] Hohwü L, Borre M, Ehlers L, Venborg Pedersen K. A short-term cost-effectiveness study comparing robot-assisted laparoscopic and open retropubic radical prostatectomy. J Med Econ. 2011;14:403-9.
[28] Hong H, Mel L, Taylor J, Wu Q, Reeves H. Effects of robotic-assisted laparoscopic prostatectomy on surgical pathology specimens. Diagn Pathol. 2012;7:24.
[29] İnkaya A, Tahra A, Sobay R, Kumcu A, Küçük EV, Boylu U. Comparison of surgical, oncological, and functional outcomes of robot-assisted and laparoscopic radical prostatectomy in patients with prostate cancer. Turk J Urol. 2019;45:410-7.
[30] Jacobsen NE, Moore KN, Estey E, Voaklander D. Open versus laparoscopic radical prostatectomy: a prospective comparison of postoperative urinary incontinence rates. J Urol. 2007;177:615-9.
[31] Johnson I, Ottosson F, Diep LM, Berg RE, Hoff JR, Wessel N, et al. Switching from laparoscopic radical prostatectomy to robot assisted laparoscopic prostatectomy: comparing oncological outcomes and complications. Scand J Urol. 2018;52:116-21.
[32] Joseph JV, Vicente I, Madeb R, Erturk E, Patel HR. Robot-assisted vs pure laparoscopic radical prostatectomy: are there any differences? BJU Int. 2005;96:39-42.
[33] Jurczok A, Zacharias M, Wagner S, Hamza A, Fornara P. Prospective non-randomized evaluation of four mediators of the systemic response after extraperitoneal laparoscopic and open retropubic radical prostatectomy. BJU Int. 2007;99:1461-6.
[34] Kasraeian A, Barret E, Chan J, Sanchez-Salas R, Validire P, Cathelineau X, et al. Comparison of the rate, location and size of positive surgical margins after laparoscopic and robot-assisted laparoscopic radical prostatectomy. BJU Int. 2011;108:1174-8.

[35] Kim SC, Song C, Kim W, Kang T, Park J, Jeong IG, et al. Factors determining functional outcomes after radical prostatectomy: robot-assisted versus retropubic. Eur Urol. 2011;60:413-9.
[36] Koizumi A, Narita S, Nara T, Takayama K, Kanda S, Numakura K, et al. Incidence and location of positive surgical margin among open, laparoscopic and robot-assisted radical prostatectomy in prostate cancer patients: a single institutional analysis. Jpn J Clin Oncol. 2018;48:765-70.
[37] Kordan Y, Barocas DA, Altamar HO, Clark PE, Chang SS, Davis R, et al. Comparison of transfusion requirements between open and robotic-assisted laparoscopic radical prostatectomy. BJU Int. 2010;106:1036-40.
[38] Krambeck AE, DiMarco DS, Rangel LJ, Bergstralh EJ, Myers RP, Blute ML, et al. Radical prostatectomy for prostatic adenocarcinoma: a matched comparison of open retropubic and robot-assisted techniques. BJU Int. 2009;103:448-53.
[39] Lo KL, Ng CF, Lam CN, Hou SS, To KF, Yip SK. Short-term outcome of patients with robot-assisted versus open radical prostatectomy: for localised carcinoma of prostate. Hong Kong Med J. 2010;16:31-5.
[40] Loeb S, Epstein JI, Ross AE, Schultz L, Humphreys EB, Jarow JP. Benign prostate glands at the bladder neck margin in robotic vs open radical prostatectomy. BJU Int. 2010;105:1446-9.
[41] Luciani LG, Mattevi D, Mantovani W, Cai T, Chiodini S, Vattovani V, et al. Retropubic, Laparoscopic, and Robot-Assisted Radical Prostatectomy: A Comparative Analysis of the Surgical Outcomes in a Single Regional Center. Curr Urol. 2017;11:36-41.
[42] Ludovico GM, Dachille G, Pagliarulo G, D'Elia C, Mondaini N, Gacci M, et al. Bilateral nerve sparing robotic-assisted radical prostatectomy is associated with faster continence recovery but not with erectile function recovery compared with retropubic open prostatectomy: the need for accurate selection of patients. Oncol Rep. 2013;29:2445-50.
[43] Magheli A, Busch J, Leva N, Schrader M, Deger S, Miller K, et al. Comparison of surgical technique (open vs. laparoscopic) on pathological and long term functional outcomes following radical prostatectomy. BMC Urol. 2014;14:18.
[44] Magheli A, Gonzalgo ML, Su LM, Guzzo TJ, Netto G, Humphreys EB, et al. Impact of surgical technique (open vs laparoscopic vs robotic-assisted) on pathological and biochemical outcomes following radical prostatectomy: an analysis using propensity score matching. BJU Int. 2011;107:1956-62.
[45] Martínez-Holguín E, Herranz-Amo F, Hernández-Cavieres J, Lledó-García E, Subirá-Ríos D, Renedo-Villar T, et al. Laparoscopic radical prostatectomy compared to open radical prostatectomy: Comparison between surgical time, complications and length of hospital stay. Actas Urol Esp. 2020;44:41-8.
[46] Martínez-Holguín E, Herranz-Amo F, Lledó-García E, Ruiz-Bel J, Esteban-Labrador L, Subirá-Ríos D, et al. Comparison between laparoscopic and open prostatectomy: Postoperative urinary continence analysis. Actas Urol Esp. 2020;44:535-41.

[47] Menon M, Shrivastava A, Tewari A, Sarle R, Hemal A, Peabody JO, et al. Laparoscopic and robot assisted radical prostatectomy: establishment of a structured program and preliminary analysis of outcomes. J Urol. 2002;168:945-9.
[48] Mirza M, Art K, Wineland L, Tawfik O, Thrasher JB. A comparison of radical perineal, radical retropubic, and robot-assisted laparoscopic prostatectomies in a single surgeon series. Prostate Cancer. 2011;2011:878323.
[49] Nadler RB, Casey JT, Zhao LC, Navai N, Smith ZL, Zhumkhawala A, et al. Is the transition from open to robotic prostatectomy fair to your patients? A single-surgeon comparison with 2-year follow-up. J Robot Surg. 2010;3:201-7.
[50] Okegawa T, Omura S, Samejima M, Ninomiya N, Taguchi S, Nakamura Y, et al. Laparoscopic radical prostatectomy versus robot-assisted radical prostatectomy: comparison of oncological outcomes at a single center. Prostate Int. 2020;8:16-21.
[51] Ong WL, Evans SM, Spelman T, Kearns PA, Murphy DG, Millar JL. Comparison of oncological and health-related quality of life outcomes between open and robot-assisted radical prostatectomy for localised prostate cancer - findings from the population-based Victorian Prostate Cancer Registry. BJU Int. 2016;118:563-9.
[52] Ou YC, Yang CR, Wang J, Cheng CL, Patel VR. Comparison of robotic-assisted versus retropubic radical prostatectomy performed by a single surgeon. Anticancer Res. 2009;29:1637-42.
[53] Papachristos A, Basto M, Te Marvelde L, Moon D. Laparoscopic versus robotic-assisted radical prostatectomy: an Australian single-surgeon series. ANZ J Surg. 2015;85:154-8.
[54] Park B, Kim W, Jeong BC, Jeon SS, Lee HM, Choi HY, et al. Comparison of oncological and functional outcomes of pure versus robotic-assisted laparoscopic radical prostatectomy performed by a single surgeon. Scand J Urol. 2013;47:10-8.
[55] Philippou P, Waine E, Rowe E. Robot-assisted laparoscopic prostatectomy versus open: comparison of the learning curve of a single surgeon. J Endourol. 2012;26:1002-8.
[56] Ploussard G, de la Taille A, Moulin M, Vordos D, Hoznek A, Abbou CC, et al. Comparisons of the perioperative, functional, and oncologic outcomes after robot-assisted versus pure extraperitoneal laparoscopic radical prostatectomy. Eur Urol. 2014;65:610-9.
[57] Porpiglia F, Morra I, Lucci Chiarissi M, Manfredi M, Mele F, Grande S, et al. Randomised controlled trial comparing laparoscopic and robot-assisted radical prostatectomy. Eur Urol. 2013;63:606-14.
[58] Punnen S, Meng MV, Cooperberg MR, Greene KL, Cowan JE, Carroll PR. How does robot-assisted radical prostatectomy (RARP) compare with open surgery in men with high-risk prostate cancer? BJU Int. 2013;112:E314-20.
[59] Qi F, Wang S, Xu H, Gao Y, Cheng G, Hua L. A comparison of perioperative outcome between robot-assisted and laparoscopic radical prostatectomy: experience of a single institution. Int Braz J Urol. 2019;45:695-702.

[60] Rocco B, Matei DV, Melegari S, Ospina JC, Mazzoleni F, Errico G, et al. Robotic vs open prostatectomy in a laparoscopically naive centre: a matched-pair analysis. BJU Int. 2009;104:991-5.
[61] Rozet F, Jaffe J, Braud G, Harmon J, Cathelineau X, Barret E, et al. A direct comparison of robotic assisted versus pure laparoscopic radical prostatectomy: a single institution experience. J Urol. 2007;178:478-82.
[62] Schroeck FR, Sun L, Freedland SJ, Albala DM, Mouraviev V, Polascik TJ, et al. Comparison of prostate-specific antigen recurrence-free survival in a contemporary cohort of patients undergoing either radical retropubic or robot-assisted laparoscopic radical prostatectomy. BJU Int. 2008;102:28-32.
[63] Silberstein JL, Su D, Glickman L, Kent M, Keren-Paz G, Vickers AJ, et al. A case-mix-adjusted comparison of early oncological outcomes of open and robotic prostatectomy performed by experienced high volume surgeons. BJU Int. 2013;111:206-12.
[64] Simsir A, Kizilay F, Aliyev B, Kalemci S. Comparison of robotic and open radical prostatectomy: Initial experience of a single surgeon. Pak J Med Sci. 2021;37:167-74.
[65] Sirisopana K, Jenjitranant P, Sangkum P, Kijvikai K, Pacharatakul S, Leenanupun C, et al. Perioperative outcomes of robotic-assisted laparoscopic radical prostatectomy, laparoscopic radical prostatectomy and open radical prostatectomy: 10 years of cases at Ramathibodi Hospital. Transl Androl Urol. 2019;8:467-75.
[66] Smith JA, Jr., Chan RC, Chang SS, Herrell SD, Clark PE, Baumgartner R, et al. A comparison of the incidence and location of positive surgical margins in robotic assisted laparoscopic radical prostatectomy and open retropubic radical prostatectomy. J Urol. 2007;178:2385-9; discussion 9-90.
[67] Sooriakumaran P, Pini G, Nyberg T, Derogar M, Carlsson S, Stranne J, et al. Erectile Function and Oncologic Outcomes Following Open Retropubic and Robot-assisted Radical Prostatectomy: Results from the LAParoscopic Prostatectomy Robot Open Trial. Eur Urol. 2018;73:618-27.
[68] Stolzenburg JU, Holze S, Neuhaus P, Kyriazis I, Do HM, Dietel A, et al. Robotic-assisted Versus Laparoscopic Surgery: Outcomes from the First Multicentre, Randomised, Patient-blinded Controlled Trial in Radical Prostatectomy (LAP-01). Eur Urol. 2021.
[69] Tewari A, Srivasatava A, Menon M. A prospective comparison of radical retropubic and robot-assisted prostatectomy: experience in one institution. BJU Int. 2003;92:205-10.
[70] Tomaszewski JJ, Matchett JC, Davies BJ, Jackman SV, Hrebinko RL, Nelson JB. Comparative hospital cost-analysis of open and robotic-assisted radical prostatectomy. Urology. 2012;80:126-9.
[71] Tozawa K, Yasui T, Umemoto Y, Mizuno K, Okada A, Kawai N, et al. Pitfalls of robot-assisted radical prostatectomy: a comparison of positive surgical margins between robotic and laparoscopic surgery. Int J Urol. 2014;21:976-9.
[72] Truesdale MD, Lee DJ, Cheetham PJ, Hruby GW, Turk AT, Badani KK. Assessment of lymph node yield after pelvic lymph node dissection in men with prostate cancer: a comparison between robot-assisted radical prostatectomy and open radical prostatectomy in the modern era. J Endourol.

2010;24:1055-60.
[73] Uvin P, de Meyer JM, Van Holderbeke G. A comparison of the peri-operative data after open radical retropubic prostatectomy or robotic-assisted laparoscopic prostatectomy. Acta Chir Belg. 2010;110:313-6.
[74] Wang R, Wood Jr DP, Hollenbeck BK, Li AY, He C, Montie JE, et al. Risk factors and quality of life for post-prostatectomy vesicourethral anastomotic stenoses. Urology. 2012;79:449-57.
[75] White MA, De Haan AP, Stephens DD, Maatman TK, Maatman TJ. Comparative analysis of surgical margins between radical retropubic prostatectomy and RALP: are patients sacrificed during initiation of robotics program? Urology. 2009;73:567-71.
[76] Williams SB, Chen MH, D'Amico AV, Weinberg AC, Kacker R, Hirsch MS, et al. Radical retropubic prostatectomy and robotic-assisted laparoscopic prostatectomy: likelihood of positive surgical margin(s). Urology. 2010;76:1097-101.
[77] Willis DL, Gonzalgo ML, Brotzman M, Feng Z, Trock B, Su LM. Comparison of outcomes between pure laparoscopic vs robot-assisted laparoscopic radical prostatectomy: a study of comparative effectiveness based upon validated quality of life outcomes. BJU Int. 2012;109:898-905.
[78] Wood DP, Schulte R, Dunn RL, Hollenbeck BK, Saur R, Wolf JS, Jr., et al. Short-term health outcome differences between robotic and conventional radical prostatectomy. Urology. 2007;70:945-9.
[79] Yaxley JW, Coughlin GD, Chambers SK, Occhipinti S, Samaratunga H, Zajdlewicz L, et al. Robot-assisted laparoscopic prostatectomy versus open radical retropubic prostatectomy: early outcomes from a randomised controlled phase 3 study. Lancet. 2016;388:1057-66.
[80] Yun JE, Lee NR, Kwak C, Rha KH, Seo SI, Hong SH, et al. Clinical outcomes and costs of robotic surgery in prostate cancer: a multiinstitutional study in Korea. Prostate Int. 2019;7:19-24.

Supplement Table S1. Characteristics of included studies

Study	Study design	Country	Sample size (R)	Sample size (L)	Sample size (O)	Age, years (RARP)	Age, years (LRP)	Age, years (ORP)	Surgical technique/approach (RARP)	Surgical technique/approach (LRP)	Surgical technique/approach (ORP)	Outcomes
Ahlering 2004	Retrospective cohort study	Unite states	60		60	62.9 (43-78)		62.7 (50 - 78)	Laparoscopic RP using the da Vinci interface		ORPs using the standard retropubic approach	Positive margin, total complication
Asimakopoulos 2011	Randomized controlled trial	Italy	64	64		61.1 ± 5.1	59.6 ± 5.4		Transperitoneal, antegrade nerve-sparing intrafascial	Transperitoneal, antegrade nerve-sparing intrafascial		Incontinence, positive margin, potency, total complication
Barocas 2010	Retrospective cohort study	Unite states	1413		491	61 ± 7.3		62 ± 7.3	Standard techniques with small modifications on 1 of 3 da Vinci®	Standard techniques with small		BCR, positive margin
Breyer 2010	Prospective cohort study	Unite states	293		695	59.7 ± 7.11		59.2 ± 6.66	Three arm da Vinci Robotic System		ORP (retropubic) was performed through a 10 cm low, midline incision	Positive margin
Carlsson 2010	Prospective cohort study	Sweden	1253		485	62 (35 - 78)		63 (47 - 77)	Karolinska prostatectomy: a robot assisted laparoscopic radical prostatectomy technique		The ORP entailed a modification of Walsh “anatomical radical retropubic prostatectomy."	Major complication, total complication
Chan 2008	Retrospective cohort study	Unite states	660		340	NR		NR	NR		NR	Positive margin
Chan 2017	Retrospective cohort study	Taiwan	40	100		65.2 ± 6.5	64.5 ± 5.9		Transperitoneal approach generally followed the Vattikuti Institute prostatectomy technique	Transperitoneal antegrade approach		BCR, estimated blood loss, incontinenece, major complication, operating time, positive margin
Choo 2013	Retrospective cohort study	South Korea	77		176	66 ± 7.7		67 ± 6.3	Transperitoneal antegrade approach		Modified Walsh anatomical retropubic ORP	BCR, estimated blood loss, incontinenece, operating time, positive margin, potency
Coronato 2009	Retrospective cohort study	Unite states	98		57	58.9		59.4	Robotic prostatectomy		Retropubic prostatectomy	Positive margin
Coughlin 2018	Randomized controlled trial	Australia	163		163	NR		NR	NR		NR	BCR, incontinenece, potency
D'Alonzo 2009	Retrospective cohort study	Unite states	78		16	59 ± 6.6		60 ± 6.9	Robot assisted laparoscopic prostatectomy		Radical retropubic prostatectomy	Estimated blood loss, operating time
Dahl 2009	Prospective cohort study	Unite states		104	102	59.5	59.9			NR	NR	BCR, major complication, positive margin, total complication
Di Pierro 2011	Prospective cohort study	Switzerla nd	75		75	62.8 (58.4 - 7.0)	64.3 (59.1 -68.0)		Transperitoneal approach, with preservation of the bladder neck and neurovascular bundles through a tension- and energyfree technique		NR	BCR, incontinenece, major complication, operating time, positive margin, potency, total complication

Doumerc 2010	Prospective cohort study	Australia	212		502	61.3 (41 - 76)		60.1 (40 - 78)	Transperitoneal surgical approach		Via infra umbilical incision	Major complication, positive margin
Drouin 2009	Retrospective cohort study	France	71	85	83	60.4 (46 - 70)	61.8 (39 - 73)	60.5 (45 - 81)	Three arm da Vinci surgical system™ and a transperitoneal approach with a six port technique	Transperitoneal approach	ORP was performed according to the anatomical technique	BCR, estimated blood loss, major complication, operating time, positive margin, total complication
Farnham 2006	Prospective cohort study	Unite states	176		103	59±7		60±7.8	NR		NR	Estimated blood loss
Ficarra 2009	Prospective cohort study	Italy	103		105	61 (57 - 67)		65 (61 - 69)	The ports for the three arm robot and the traditional laparoscopic tools were placed		The procedure was extraperitoneal through an infraumbilical pubic incision, with a standard iliac and obturator lymph node dissection in every patient.	Incontinenece, positive margin, potency, total complication
Forsmark 2018	Prospective cohort study	Sweden	1835		803	63		64	NR		NR	Operating time
Fracalanza 2008	Prospective cohort study	Italy	35		26	62 (56 - 68)		68.5 (59 - 71)	da Vinci system; performed with transperitoneal approach with an antegrade prostatic dissection		Walsh technique	Operating time, positive margin
Froehner 2013	Retrospective cohort study	Germany	252		1925	62.8		65.2	3-arm da Vinci robotic device		The retropubic approach	Positive margin
Froehner 2013	Retrospective cohort study	Germany	317		2437	NR		NR	3-arm da Vinci robotic device		The retropubic approach	Total complication
Guazzoni 2006	Randomized controlled trial	Italy		60	60		62.99 ± 8.2	62.9 ± 7.4		The trans-peritoneal route according to the Montsouris technique	Walsh technique	Estimated blood loss, operating time, positive margin
Haese 2019	Prospective cohort study	Germany	3783		7007	65 (59 - 69)		64 (58 - 68)	NR		NR	BCR, incontinenece, positive margin, potency
Haglind 2015	Prospective cohort study	Sweden	1847		778	63 (58-66)		63 (59-67)	NR		NR	Incontinenece, positive margin, potency
Hakimi 2009	Prospective cohort study	Unite states	81	124		59.8 (42-71)	59.6 (43-72)		NR	NR		BCR, incontinenece, major complication, positive margin, potency, total complication
Ham 2008	Prospective cohort study	South Korea	188		110	67.3 ± 6.2		66.9 ± 6.0	Standard retropubic approach and RP by the transperitoneal approach using four robotic arms		Standard retropubic approach	Incontinenece, positive margin, potency
Hohwü 2011	Retrospective cohort study	Denmark	77		154	62.2		62.2	NR		Conventional RRP	BCR, incontinenece, potency

Hong 2012	Retrospective cohort study	Unite states	182		80	60.8 ± 6.8		60.5 ± 6.8	3-arm da Vinci robotic device		Conventional RRP	Positive margin
İnkaya 2019	Retrospective cohort study	Turkey	778	48		62.3 ± 6.5	63.82 ± 5.8		Transperitoneal approach	Transperitoneal approach		BCR, estimated blood loss, incontinenece, major complication, operating time, positive margin, potency
Jacobsen 2007	Prospective cohort study	Canada		67	172		62.3 ± 6.4, 60.9 ± 6.6	63.7 ± 5.7		The trans-peritoneal route according to the Montsouris technique	Open radical retropubic prostatectomy	Incontinenece, positive margin
Johnson 2018	Prospective cohort study	Norway	1081	544		64 (40-76)	62 (42-76)		Montsouris transperitoneal/transvescical	The trans-peritoneal route according to the Montsouris technique		Major complication, operating time, potency, total complication
Joseph 2005	Retrospective cohort study	Unite states/France	50	50		59.6 ± 1.6	61.8 ± 1.6		Extraperitoneal approach	Extraperitoneal approach		Estimated blood loss, incontinenece, operating time
Jurczok 2007	Prospective cohort study	Germany		163	240		62.9 (42-74)	64.8 (52-76)		The pre-peritoneal technique with a pelvic lymphadenectomy	RRP (ascending) with pelvic lymph node dissection	Positive margin, total complication
Kasraeian 2011	Retrospective cohort study	France	200	200		60.8 (44 - 73)	61.9 (45 - 75)		Extraperitoneal interfascial technique	Extraperitoneal interfascial technique		Positive margin
Kim 2011	Prospective cohort study	South Korea	528		235	64.2 ± 7.3		66.5 ± 5.7	Transperitoneal antegrade approach		Extraperitoneal retrograde approach	Positive margin
Koizumi 2018	Retrospective cohort study	Japan	100	100	100	68.0 (50-76)	66.0 (53-76)	68.0 (42-81)	Transperitoneal posterior approach using the da Vinci S Robotic System	NR	NR	Positive margin
Kordan 2010	Prospective cohort study	Unite states	830		414	60.5 ± 7.2		61.5 ± 7.5	Robotic assisted laparoscopic RP		Open retropubic RP	Positive margin
Krambeck 2009	Retrospective cohort study	Unite states	294		588	61 (38.0 - 76.0)		61 (41.0 - 77.0)	da Vinci system using modified variations of previously reported techniques		Radical retropubic prostatectomy	BCR, incontinenece, positive margin, potency, total complication
Lo 2010	Retrospective cohort study	Hong Kong	20		20	64 (52 - 75)		66 (47 - 76)	Robotic radical prostatectomy, the 6 port transperitoneal VIP technique		Extraperitoneal retropubic approach	BCR, incontinenece, operating time, positive margin
Loeb 2010	Prospective cohort study	Unite states	152		137	58.1 ± 5.6			Various techniques but the prostatic dissection was always antegrade with division of the bladder neck from anterior and posterior		Standard anatomical fashion described by Latiff and Gomez	BCR, positive margin
Luciani 2017	Retrospective cohort study	Italy	100	100	100	66 (63 - 71)	69 (64 - 73)	72 (69 - 77)	NR	NR	NR	Major complication, positive margin, total complication

Ludovico 2013	Prospective cohort study	Italy	82		48	68.1 ± 2.3		66.7 ± 9.9	Transperitoneally, dissecting the anterolateral surface of the prostate using an intrafascial technique		Performed as described by Walsh in 1998	Incontinenece, major complication, positive margin, potency, total complication
Magheli 2014	Prospective cohort study	Germany		171	168		62.3 ± 5.7	62.6 ± 5.4		NR	NR	Incontinenece, positive margin, potency
Magheli 2011	Retrospective cohort study	Unite states	522	522	522	58.3 ± 6.3	58.4 ± 6.4	58.8 ± 6.1	NR	NR	Conventional RRP	BCR, positive margin
Martínez-Holguín 2020	Retrospective cohort study	Spain		206	312		64<br>(44 - 76)	63<br>(58 - 68)		NR	NR	Major complication, positive margin, total complication
Martínez-Holguín 2020	Retrospective cohort study	Spain		206	312		64<br>(44 - 76)	63<br>(58 - 68)		NR	NR	Incontinenece
Menon 2002	Prospective cohort study	Unite states	40	40		62.8 ± 1.1	60.7 ± 1.2		Montsouris transperitoneal	Montsouris transperitoneal		Estimated blood loss, operating time, positive margin
Mirza 2011	Retrospective cohort study	Unite states	191		180	60.1 ± 7.3		61.7 ± 6.8	NR		NR	Positive margin
Nadler 2010	Retrospective cohort study	Unite states	50		50	59.7<br>(44 - 77)		60.0<br>(40 - 75)	Four arm, five port technique		Performed as described by McCarthy and Catalona	BCR, incontinenece, positive margin, potency, total complication
Okegawa 2020	Prospective cohort study	Japan	450	250		66<br>(48 - 82)	68<br>(51 - 76)		Montsouris transperitoneal	Montsouris transperitoneal		BCR, positive margin
Ong 2016	Prospective cohort study	Australia	885		1117	62.1 ± 6.7		62.3 ± 6.7	NR		NR	BCR, incontinenece, potency, positive margin
Ou 2009	Retrospective cohort study	Taiwan of China	30		30	67.3 ± 6.2		70.0 ± 6.1	Performed as described by Patel (13, 14) with minor modifications		Retropubic radical prostatectomy using Walsh's technique	BCR, estimated blood loss, incontinenece, operating time, positive margin, potency
Papachristos 2015	Retrospective cohort study	Australia	100	100		60.5<br>(45 - 75)	62.5<br>(45 - 72)		Transperitoneal	Extraperitoneal		BCR, incontinenece, major complication, positive margin, potency, total complication
Park 2013	Retrospective cohort study	South Korea	183	144		63<br>(44 - 75)	67<br>(38 - 77)		NR	NR		BCR, incontinenece, positive margin, potency, total complication
Philippou 2012	Retrospective cohort study	United Kingdom	50		50	62.4 ± 5.6		62.5 ± 6.4	Transperitoneal six-port antegrade approach		According to the principles described by Walsh (1983)	BCR, estimated blood loss, incontinenece, major complication, operating time, positive margin, potency, total complication

Ploussard 2014	Prospective cohort study	France	1009	1377		62.7	62.7		Transperitoneal antegrade approach	Transperitoneal antegrade approach		BCR, incontinenece, major complication, positive margin, potency, total complication
Porpiglia 2013	Randomized controlled trial	Italy	60	60		63.9 ± 6.7	64.7 ± 5.9		Transperitoneal antegrade approach	Transperitoneal antegrade approach		BCR, estimated blood loss, incontinenece, operating time, positive margin, potency, total complication
Punnen 2013	Retrospective cohort study	Unite states	233		177	61.3 ± 6.78		60.8 ± 6.38	NR		Standard fashion via retrograde dissection of the prostate gland	BCR, estimated blood loss, positive margin
Qi 2019	Retrospective cohort study	China	132	78		72.5 (56-80)<br>66 (48-79)	69 (68 - 81)		Transperitoneal or extraperitoneal approach	Extraperitoneal approach		Positive margin
Rocco 2009	Retrospective cohort study	Italy	120		240	63 (47 - 76)		63 (46 - 77)	Patel technique		Walsh technique	Incontinenece, positive margin, potency
Rozet 2007	Prospective cohort study	France	133	133		62.0 (49 - 76)	62.5 (47 - 74)		Extraperitoneal	Extraperitoneal		Positive margin, total complication
Schroeck 2008	Retrospective cohort study	Unite states	362		435	59.2 (54.5-63.8)		60.3 (55.3-64.7)	da Vinci system; performed using Vattikuti Institute technique		Using a standard retropubic approach	BCR, positive margin
Silberstein 2013	Retrospective cohort study	Unite states	493		961	60 (54 - 65)		61 (56 - 66)	Performed with modifications to previously described by Lowrance (2012)		Performed with modifications to previously described by Walsh (1998)	BCR, positive margin
Simsir 2021	Retrospective cohort study	Turkey	100		100	64.6 (47 - 79)		62.8 (48 - 76)	The Montsouris technique		Walsh technique	BCR, incontinenece, major complication, positive margin, potency, total complication
Sirisopana 2019	Retrospective cohort study	Thailand	295	241	128	68 (63 - 72)	68 (63 - 72)	68 (62 - 73)	Intraperitoneal fashion using the da Vinci Surgical System Si	NR	Retropubic fashion	Major complication, positive margin, total complication
Smith 2007	Retrospective cohort study	Unite states	200		200	60.3 ± 7.3		61.1 ± 7.35	RALP a 5 port technique		Standard fashion via an infraumbilical midline incision	Positive margin
Sooriakumaran 2018	Prospective cohort study	Sweden	1792		753	63.3 (58.4-66.9)		63.5 (59.3-67.3)	NR		NR	BCR, positive margin, potency
Stolzenburg 2021	Randomized controlled trial	Germany	547	171		65 (59 - 69)	65 (59 - 70)		Transperitoneal/extraperitoneal approach	Transperitoneal/extraperitoneal approach		Incontinenece, major complication, positive margin, potency,total complication
Tewari 2003	Prospective cohort study	Unite states	200		100	59.9 (40 - 72)		63.1 (42.8 - 72)	da Vinci system (robotically assisted Vattikuti Institute prostatectomy)		Anatomical technique and VIP	BCR, major complication, positive margin, total complication

Tomaszewski 2012	Retrospective cohort study	Belgium	115		358	NR		NR	NR		Conventional RRP	Operating time
Tozawa 2014	Retrospective cohort study	Japan	157	551		67.0 (41 - 78)	67.4 (47 - 82)		The modified Montsouris method using the four-armda Vinci S Robotic System	NR		Positive margin
Truesdale 2010	Retrospective cohort study	Unite states	99		217	59.2 ± 7.1		61.7 ± 6.8	RARP, the PLND was performed via the identical port siteconfiguration used for prostatectomy		ORP, PLND was performed via the lower abdominal midline incision for prostatectomy	Estimated blood loss, operating time
Uvin 2010	Retrospective cohort study	Belgium	13		9	62.6		65.7	da Vinci robotic device		Conventional RRP	Positive margin
Wang 2012	Retrospective cohort study	Unite states	1038		707	60.4		59.7	Intraperitoneal or extraperitoneal approach		Conventional RRP	BCR, positive margin
White 2009	Retrospective cohort study	Unite states	50		50	62		64.7	Technique as described by Menon		Performed in the traditional fashion	Positive margin
Williams 2010	Prospective cohort study	Unite states	604		346	NR		NR	Transperitoneal retrograde approach		Radical retropubic prostatectomy	Positive margin
Willis 2012	Prospective cohort study	Unite states	121	161		58.1 ± 6.5	58.2 ± 7.9		Montsouris retrovesical approach to prostatectomy	Montsouris retrovesical approach to prostatectomy		Estimated blood loss, operating time, positive margin
Wood 2007	Prospective cohort study	Unite states	165		152	60.2		59.2	Robotic radical prostatectomy		Conventional radical prostatectomy	Estimated blood loss, operating time, positive margin
Yaxley 2016	Randomized controlled trial	Australia	163		163	NR		NR	NR		NR	Estimated blood loss, major complication, operating time, positive margin, total complication
Yun 2019	Retrospective cohort study	South Korea	559	170	135	68 (63 - 73)	71 (67 - 74)	73 (68 - 77)	NR	NR	NR	BCR, positive margin, total complication

BCR, biochemical recur; LRP, laparoscopic radical prostatectomy; NR, not reported; ORP, open radical prostatectomy; PLND, pelvis lymph node dissection; RARP, robot assisted radical prostatectomy; RRP, radical retropubic prostatectomy; VIP, Vattikuti Institute prostatectomy

Supplement Table S2. Relative effect table of opreation method's efficacy for biochemical recurrence

Comparison of the included interventions: risk ratio (95% CrI). Each cell gives the effect of the column-defining intervention relative to the row-defining intervention.		
**ORP**	1.060 (0.771, 1.452)	**0.713 (0.587, 0.869)**
	**LRP**	**0.672 (0.505, 0.895)**
		**RARP**

LRP, laparoscopic radical prostatectomy; OPR, open radical prostatectomy; RARP, robotic-assisted radical prostatectomy

Supplement Table S3. Relative effect table of opreation method's efficacy for positive surgical margin

Comparison of the included interventions: risk ratio (95% CrI). Each cell gives the effect of the column-defining intervention relative to the row-defining intervention.		
**ORP**	0.878 (0.756, 1.019)	**0.893 (0.807, 0.985)**
	**LRP**	1.017 (0.886, 1.166)
		**RARP**

LRP, laparoscopic radical prostatectomy; OPR, open radical prostatectomy; RARP, robotic-assisted radical prostatectomy

Supplement Table S4. Relative effect table of opreation method's efficacy for continence

Comparison of the included interventions: risk ratio (95% CrI). Each cell gives the effect of the column-defining intervention relative to the row-defining intervention.		
**ORP**	0.921 (0.845, 1.007)	1.057 (0.997, 1.124)
	**LRP**	**1.148 (1.064, 1.237)**
		**RARP**

LRP, laparoscopic radical prostatectomy; OPR, open radical prostatectomy; RARP, robotic-assisted radical prostatectomy

Supplement Table S5. Relative effect table of opreation method's efficacy for potency

Comparison of the included interventions: risk ratio (95% CrI). Each cell gives the effect of the column-defining intervention relative to the row-defining intervention.		
**ORP**	0.836 (0.657, 1.065)	**1.201 (1.047, 1.402)**
	**LRP**	**1.438 (1.191, 1.762)**
		**RARP**

LRP, laparoscopic radical prostatectomy; OPR, open radical prostatectomy; RARP, robotic-assisted radical prostatectomy

Supplement Table S6. Relative effect table of opreation method's efficacy for estimated blood loss

Comparison of the included interventions: risk ratio (95% CrI). Each cell gives the effect of the column-defining intervention relative to the row-defining intervention.		
**ORP**	-1,340.359 (-4,128.900, 1,420.257)	-1,662.253 (-3,597.514, 315.091)
	**LRP**	-316.020 (-2,576.738, 1,998.387)
		**RARP**

LRP, laparoscopic radical prostatectomy; OPR, open radical prostatectomy; RARP, robotic-assisted radical prostatectomy

Supplement Table S7. Relative effect table of opreation method's efficacy for operation time

Comparison of the included interventions: risk ratio (95% CrI). Each cell gives the effect of the column-defining intervention relative to the row-defining intervention.		
**ORP**	**40.817 (1.615, 80.094)**	**97.092 (39.353, 152.799)**
	**LRP**	**56.362 (10.668, 102.759)**
		**RARP**

LRP, laparoscopic radical prostatectomy; OPR, open radical prostatectomy; RARP, robotic-assisted radical prostatectomy

Supplement Table S8. Relative effect table of opreation method's efficacy for total complication

Comparison of the included interventions: risk ratio (95% CrI). Each cell gives the effect of the column-defining intervention relative to the row-defining intervention.		
**ORP**	0.904 (0.572, 1.419)	**0.631 (0.440, 0.918)**
	**LRP**	0.703 (0.467, 1.034)
		**RARP**

LRP, laparoscopic radical prostatectomy; OPR, open radical prostatectomy; RARP, robotic-assisted radical prostatectomy

Supplement Table S9. Relative effect table of opreation method's efficacy for major complication

Comparison of the included interventions: risk ratio (95% CrI). Each cell gives the effect of the column-defining intervention relative to the row-defining intervention.		
**ORP**	0.826 (0.380, 1.935)	**0.493 (0.248, 0.995)**
	**LRP**	0.598 (0.294, 1.135)
		**RARP**

LRP, laparoscopic radical prostatectomy; OPR, open radical prostatectomy; RARP, robotic-assisted radical prostatectomy

Supplement Table S10. The Newcastle-Ottawa scale for non-RCT studies

Study	Selection 1	Selection 2	Selection 3	Selection 4	Comparability A	Comparability B	Exposure 1	Exposure 2	Exposure 3	Scores
Ahlering 2004	*	–	–	*	*	*	*	–	–	5
Barocas 2010	*	*	–	*	*	*	*	*	–	7
Breyer 2010	*	*	–	*	*	*	*	*	–	7
Carlsson 2010	*	*	–	*	*	*	*	*	–	7
Chan 2008	*	–	–	*	*	*	*	*	–	6
Chan 2017	*	–	–	*	*	*	*	*	–	6
Choo 2013	*	–	–	*	*	*	*	*	–	6
Coronato 2009	*	*	–	*	*	*	*	*	–	7
D'Alonzo 2009	*	–	–	*	*	*	*	*	–	6
Dahl 2009	*	*	–	*	*	*	*	*	–	7
Di Pierro 2011	*	*	–	*	*	*	*	*	–	7
Doumerc 2010	*	–	–	*	*	*	*	*	–	6

Drouin 2009	*	–	–	*	*	*	*	*	–	6
Farnham 2006	*	–	–	*	*	*	*	*	–	6
Ficarra 2009	*	*	–	*	*	*	*	*	–	7
Forsmark 2018	*	–	–	*	*	*	*	*	–	6
Fracalanza 2008	*	*	–	*	*	*	*	*	–	7
Froehner 2013	*	*	–	*	*	*	*	*	–	7
Froehner 2013	*	*	–	*	*	*	*	*	–	7
Haese 2019	*	*	–	*	*	*	*	*	–	7
Haglind 2015	*	*	–	*	*	*	*	*	–	7
Hakimi 2009	*	*	–	*	*	*	*	*	–	7
Ham 2008	*	*	–	*	*	*	*	*	–	7
Hohwü 2011	*	–	–	*	*	*	*	*	–	6
Hong 2012	*	–	–	*	*	*	*	*	–	6
İnkaya 2019	*	*	–	*	*	*	*	*	–	7

Jacobsen 2007	*	*	–	*	*	*	*	*	–	7
Johnson 2018	*	*	–	*	*	*	*	*	–	7
Joseph 2005	*	*	–	*	*	*	*	*	–	7
Jurczok 2007	*	–	–	*	*	*	*	*	–	6
Kasraeian 2011	*	–	–	*	*	*	*	–	–	5
Kim 2011	*	*	–	*	*	*	*	*	–	7
Koizumi 2018	*	–	–	*	*	*	*	–	–	5
Kordan 2010	*	–	–	*	*	*	*	*	–	6
Krambeck 2009	*	–	–	*	*	*	*	*	–	6
Lo 2010	*	*	–	*	*	*	*	*	–	7
Loeb 2010	*	*	–	*	*	*	*	*	–	7
Luciani 2017	*	–	–	*	*	*	*	*	–	6
Ludovico 2013	*	*	–	*	*	*	*	*	–	7
Magheli 2014	*	–	–	*	*	*	*	*	–	6

Magheli 2011	*	–	–	*	*	*	*	*	–	6
Martínez-Holguín 2020	*	–	–	*	*	*	*	*	–	6
Martínez-Holguín 2020	*	–	–	*	*	*	*	*	–	6
Menon 2002	*	*	–	*	*	*	*	*	–	7
Mirza 2011	*	–	–	*	*	*	*	*	–	6
Nadler 2010	*	–	–	*	*	*	*	*	–	6
Okegawa 2020	*	–	–	*	*	*	*	*	–	6
Ong 2016	*	–	–	*	*	*	*	*	–	6
Ou 2009	*	–	–	*	*	*	*	–	–	5
Papachristos 2015	*	*	–	*	*	*	*	*	–	7
Park 2013	*	–	–	*	*	*	*	–	–	5
Philippou 2012	*	–	–	*	*	*	*	*	–	6
Ploussard 2014	*	*	–	*	*	*	*	*	–	7

Punnen 2013	*	–	–	*	*	*	*	*	–	6
Qi 2019	*	*	–	*	*	*	*	*	–	7
Rocco 2009	*	–	–	*	*	*	*	*	–	6
Rozet 2007	*	–	–	*	*	*	*	*	–	6
Schroeck 2008	*	–	–	*	*	*	*	*	–	6
Silberstein 2013	*	–	–	*	*	*	*	*	–	6
Simsir 2021	*	*	–	*	*	*	*	*	–	7
Sirisopana 2019	*	–	–	*	*	*	*	*	–	6
Smith 2007	*	–	–	*	*	*	*	*	–	6
Sooriakumaran 2018	*	–	–	*	*	*	*	*	–	6
Tewari 2003	*	–	–	*	*	*	*	*	–	6
Tomaszewski 2012	*	–	–	*	*	*	*	–	–	5
Tozawa 2014	*	–	–	*	*	*	*	–	–	5
Truesdale 2010	*	–	–	*	*	*	*	*	–	6

Uvin 2010	*	–	–	*	*	*	*	–	–	5
Wang 2012	*	*	–	*	*	*	*	*	–	7
White 2009	*	–	–	*	*	*	*	*	–	6
Williams 2010	*	–	–	*	*	*	*	*	–	6
Willis 2012	*	–	–	*	*	*	*	*	–	6
Wood 2007	*	–	–	*	*	*	*	*	–	6
Yun 2019	*	–	–	*	*	*	*	*	–	6

Supplement Figure S1. The risk of bias graph for RCT

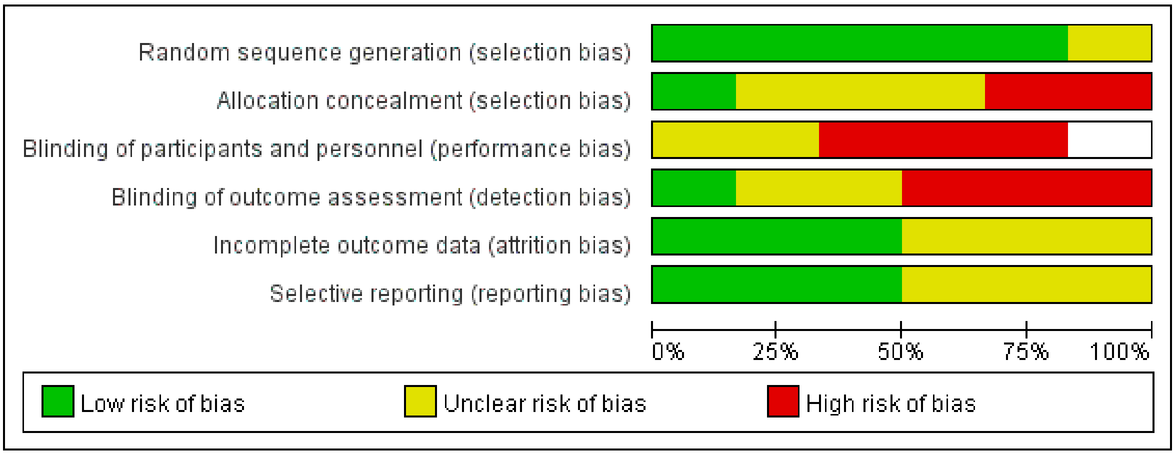

Supplement Figure S2. The risk of bias assessment for RCT

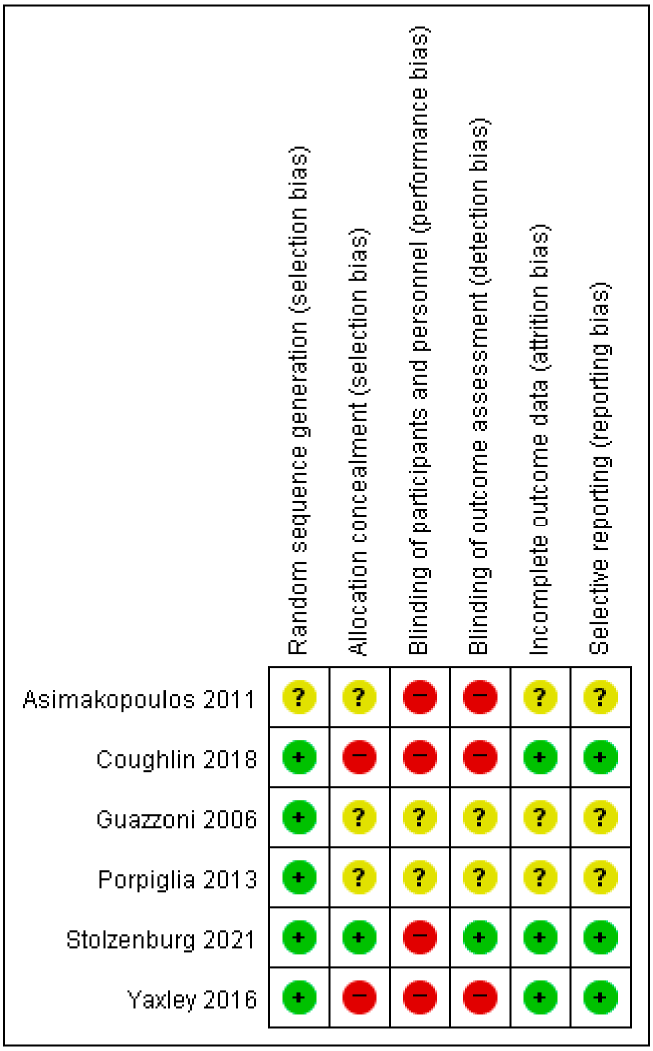

Supplement: Supplementary file 1 [file medicina-61-00061-s001.zip › medicina-3401955-supplementary.pdf]
